# Transcatheter arterial embolization for hemoptysis in children with bronchopulmonary artery fistulas: a single-center retrospective analysis

**DOI:** 10.3389/fped.2026.1822389

**Published:** 2026-09-30

**Authors:** Zhuang Liu, Liang Wang, Jing Li, Xin Zhang, Jie Zhou, Dan Song

**Affiliations:** 1 Department of Vascular Anomalies and Interventional Radiology, Children’s Hospital Affiliated to Shandong University, Jinan, Shandong, China; 2 Department of Vascular Anomalies and Interventional Radiology, Jinan Children’s Hospital, Jinan, Shandong, China

**Keywords:** bronchopulmonary artery fistula, efficacy, hemoptysis, interventional therapy, pediatric patients

## Abstract

**Objective:**

To evaluate the safety and efficacy of transcatheter interventional embolization for hemoptysis secondary to bronchopulmonary artery fistula (BPAF) in pediatric patients.

**Methods:**

A retrospective review was conducted on the clinical profiles and digital subtraction angiography (DSA) characteristics of 17 pediatric patients with BPAF-related hemoptysis. Clinical outcomes and follow-up data were analyzed.

**Results:**

The cohort comprised 11 boys and 6 girls, aged 7 months to 14 years. DSA demonstrated varying degrees of bronchopulmonary shunts. All patients successfully underwent super-selective embolization of the target vessels via a microcatheter. During hospitalization, hemoptysis was immediately resolved in 16 patients (94. 1%), while Patient No. 17 experienced persistent bleeding post-intervention and required immediate surgical treatment. During the long-term post-discharge follow-up, three patients experienced recurrence: Patient No. 12 developed recurrent hemoptysis at 2 years post-procedure, which was managed with a second and a subsequent third embolization due to another recurrence 3 months later, achieving long-term remission thereafter; Patient No. 15 experienced recurrence 1 year post-procedure and remained symptom-free after a second embolization; Patient No. 14 recurred 2 months post-procedure, and due to the inability of the microcatheter to reach the target vessel during the second angiography, the patient was successfully treated with surgery.

**Conclusion:**

Transcatheter interventional therapy is a safe and effective approach for managing BPAF-related hemoptysis in pediatric patients. For fistulas with high-volume shunts, positioning the microcatheter as close to the fistula orifice as possible for embolization may optimize technical and clinical success. Considering the relatively small sample size, the present results should be interpreted with caution and verified by larger follow-up studies.

## 1 Introduction

Hemoptysis is a critical and potentially life-threatening condition in pediatric patients, characterized by respiratory tract or pulmonary hemorrhage distal to the larynx. Clinically, its presentation varies from the expectoration of fresh blood to blood-streaked sputum. Due to an underdeveloped cough reflex and limited ability to actively expectorate sputum, hemoptysis in children often signifies more profound bleeding than clinically apparent. Accurately quantifying blood loss in pediatric practice remains challenging, and a universal gold standard for grading the severity of pediatric hemoptysis is currently lacking. In adult populations, massive hemoptysis is conventionally defined as a 24-hour blood loss of ≥ 500 mL, or an hourly bleeding rate of ≥ 100 ml (1). For pediatric patients, Batra et al. proposed that a 24-hour expectoration volume exceeding >8 ml/kg should be classified as massive hemoptysis (2). While low-volume hemoptysis can be effectively managed via etiologic treatment, systemic hemostatic agents, bronchoscopic administration of vasoconstrictors, electrocautery, or balloon tamponade, massive hemoptysis carries an imminent risk of asphyxiation and fatal exsanguination within a short window. Transcatheter embolization has long been established as a cornerstone therapy for adult hemoptysis. In recent years, driven by the rapid advancements of interventional radiology in pediatrics, transcatheter embolization has increasingly emerged as a widely recognized, first-line therapeutic strategy for the management of pediatric massive hemoptysis.

While transcatheter bronchial artery embolization for hemoptysis has been extensively investigated in adults, pediatric data remain scarce and largely restricted to isolated case reports, leaving a critical gap in the systematic analysis of bronchopulmonary artery fistula (BPAF) in children (3). To address this, this study retrospectively analyzed 17 pediatric patients with BPAF-associated hemoptysis treated at Jinan Children's Hospital between January 2016 and January 2024. By evaluating intraoperative angiographic findings, embolization parameters, and long-term follow-up outcomes, we correlated the digital subtraction angiography (DSA) characteristics with clinical prognoses. This study aims to delineate tailored embolization strategies tailored for the slender and tortuous vasculature typically encountered in pediatric patients, while elucidating the specific etiologies underlying treatment failure and recurrence, thereby providing robust clinical evidence to guide pediatric interventional therapy.

This retrospective study was conducted in accordance with the Declaration of Helsinki.

Ethical approval was waived by the Institutional Review Board (IRB) of our hospital due to the retrospective nature of the study, which only collected and analyzed anonymous existing medical records and follow- up data without any intervention on patients. Informed consent was therefore not required.

## 2 Materials and methods

### 2.1 general data and inclusion criteria

The cohort comprised 11 boys and 6 girls, with a median age ranging from 7 months to 12 years. All 17 pediatric patients presented with hemoptysis as their primary symptom warranting admission, and the diagnosis of BPAF was universally confirmed via DSA, which revealed varying degrees of shunts. The patients had no history of congenital heart disease, pulmonary arterial hypertension, or prior thoracic surgery.

### 2.2 Research

Upon admission, all patients underwent a comprehensive evaluation, including chest computed tomography (CT), electronic bronchoscopy, and laboratory assessments (complete blood count and coagulation profiles). Preoperative CT imaging should incorporate bronchial artery reconstruction to delineate the ostial anatomy of the bronchial arteries and identify potential hemorrhagic target vessels. Transcatheter angiography and embolization were performed once contraindications for interventional procedures were ruled out. The specific surgical protocol was as follows:

Under general anesthesia, the bilateral inguinal regions were routinely prepped and draped in a sterile manner. Access was established via the unilateral femoral artery (left or right) using a 4F vascular sheath. A 4F pigtail catheter was initially deployed to perform angiography of the aortic arch and descending aorta. Subsequently, a 4F Cobra catheter was utilized for selective catheterization of the bronchial and internal thoracic arteries. If abnormal parenchymal staining was detected, super-selective catheterization of the feeder vessels was achieved using a microcatheter. In this cohort, diagnostic angiography confirmed that the culprit vessels in all patients were bronchial arteries originating directly from the descending aorta. Angiographically, active bleeding was indicated by contrast agent pooling and transient stasis in the distal branches of the bronchial arteries; concurrently, a BPAF was identified by the early contrast opacification of the distal pulmonary arteries. After delineating the vascular anatomy and lesion location, the microcatheter, coaxially supported by a guidewire, was advanced as close to the fistula orifice as technically feasible. Embolization was then meticulously performed via the slow injection of polyvinyl alcohol (PVA) particles (300–500 μm) and gelatin sponge particles. The procedure was concluded only after repeat angiography confirmed the complete resolution or significant reduction of the abnormal contrast staining.

Regarding the technical approach, in one patient, the culprit feeding artery was excessively tortuous, preventing the microcatheter from being advanced super-selectively to the lesion; consequently, proximal embolization of the feeding artery was performed. For the remaining patients, super-selective embolization was successfully executed with the microcatheter advanced directly to the lesion site.

Postoperatively, patients were monitored for hemoptysis control and underwent continuous clinical follow-up after discharge. Therapeutic efficacy was evaluated based on the Clinical Treatment Guidelines for Hemoptysis using the following criteria:

- Complete Remission (Cured): Complete cessation of hemoptysis within 24 h post-procedure, with no recurrence during the 3-month follow-up.
- Partial Response (Improved): A marked reduction in both the frequency and volume of hemoptysis compared with baseline levels.
- Treatment Failure (Ineffective): No significant improvement in the frequency or total volume of hemoptysis, or an acute exacerbation of symptoms.

## 3 Results

Among the 17 patients who underwent transcatheter embolization, no significant intraoperative or postoperative complications or adverse reactions were observed (Table 1). In 16 patients, hemoptysis progressively resolved within 3 to 5 days post-procedure. However, Patient 17 experienced persistent hemoptysis immediately after the intervention and required emergency surgical resection. Following discharge, the cohort underwent continuous clinical follow-up. Recurrent hemoptysis occurred in three patients during the long-term follow-up period: Patient 12 experienced a recurrence at 2 years postoperatively and was managed with a second embolization; due to another recurrence 3 months later, a third interventional procedure was performed, which achieved long-term remission with no further recurrence. Patient 14 experienced recurrence 2 months post-procedure; during the second angiography, the microcatheter could not be advanced to the target vessel, and the patient was immediately referred for surgical treatment. Patient 15 presented with recurrent hemoptysis 1 year after the initial intervention and achieved complete symptom resolution following a second embolization. The scheduled post-procedure follow-up period for all subjects was at least 12 months, with a median follow-up of 12 months (range, 12-27 months).

**Table 1:** Clinical characteristics.

Age	Embolicmaterial	Symptom	Culpritvessel	Causesofhemoptysis	Result
7Y	PVA	Coughing for 9 days, hemoptysis twice	Thyrocervical trunk、Right bronchial artery	Pneumonia	Recovery
3 years and 7 months	PVA	Fever for 3 days, hemoptysis once	Right intercostal-bronchial trunk、Right bronchial artery	Pneumonia	Recovery
8Y	PVA	40 days of atelectasis and 3 days of hemoptysis	Right intercostal-bronchial trunk、Left and right bronchial arteries、Left bronchial artery	Pneumonia	Recovery
8Y	PVA	Pneumonia and atelectasis for 1 month	Right intercostal-bronchial trunk 、Common trunk of the left and right bronchial arteries	Pneumonia	Recovery
7Y	PVA	Coughing for half a month, hemoptysis for one day	Common trunk of the left and right bronchial arteries、Intercostal-bronchial trunk	Pneumonia	Recovery
14Y	PVA	Hemoptysis for 4 h	Right bronchial artery	Vascular anomaly	Recovery
6Y	PVA	Nasal bleeding and phlegm blood for 3 months	Common trunk of the left and right bronchial arteries、Intercostal-bronchial trunk	Vascular anomaly	Recovery
2Y	PVA	Hemoptysis for 5 days	Left subclavian artery、Common trunk of the left and right bronchial arteries	Pneumonia	Recovery
14Y	PVA	Cough with stagnant blood for 3 days	Right bronchial artery	Pneumonia	Recovery
2 years and 9 months	PVA，Gelatin sponge	Hemoptysis for 3 days	Right intercostal-bronchial trunk、Common trunk of the left and right bronchial arteries	Vascular anomaly	Recovery
5 years and 4 months	PVA	Hemoptysis for 1 day	Common trunk of the left and right bronchial arteries、left subclavian artery	Pneumonia、Bronchial Dieulafoy disease	Recovery
13Y	PVA，Gelatin sponge	Hemoptysis for 6 days	left subclavian artery、Right internal mammary artery、Right phrenic artery	Vascular anomaly	No recurrence after 3 interventional treatments
10Y	PVA	Hemoptysis for 6 h	Common trunk of the left and right bronchial arteries	Vascular anomaly	Recovery
4Y	PVA	Hemoptysis for 3 days	Right bronchial artery	Vascular anomaly	Surgical resection after the second angiography
12Y	PVA	Coughing for 8 days, intermittent hemoptysis for 3 days	Right internal mammary artery、Right phrenic artery、Right intercostal-bronchial trunk、Common trunk of the left and right bronchial arteries	Pneumonia、Vascular anomaly	After 1 year of recurrence of hemoptysis, receive interventional treatment again
7 months	PVA	Coughing for 13 days, hemoptysis once	Common trunk of the left and right bronchial arteries、Right inferior phrenic artery、Right internal mammary artery	Pneumonia	Recovery
3Y	PVA	Coughing for 1 month, hemoptysis for 21 days	Left internal mammary artery、Common trunk of the left and right bronchial arteries	Pulmonary vascular dysplasia	Surgical resection immediately after intervention surgery

## 4 Discussion

### 4.1 Etiology and mechanism of hemoptysis

Compared with adult populations, the etiology of hemoptysis in pediatric patients is substantially more complex and heavily influenced by age, geographic region, and localized healthcare capabilities. Distinct epidemiological variations exist between China and Western countries: in Western cohorts, the primary etiologies of pediatric hemoptysis encompass lower respiratory tract infections, followed by bronchiectasis, congenital heart disease, and foreign body aspiration (4–8). In contrast, in China, idiopathic pulmonary hemosiderosis (IPH) represents the leading cause, followed by respiratory infections, pulmonary vascular malformations, congenital heart disease, and neoplasms (8–10). In the current study, Patient 17 presented with a structural anomaly of the left lung, which resulted in a hypoplastic left pulmonary vein and partial pulmonary vein occlusion. The underlying mechanism triggering hemoptysis in this specific case was localized pulmonary congestion.

The lungs feature a dual vascular supply, with the pulmonary circulation contributing 99% of the total blood volume and the bronchial circulation accounting for the remaining 1%. While the pulmonary artery primarily facilitates gas exchange, the bronchial artery provides essential nutritional support to the airway structures (11). Given that the bronchial arteries arise from the systemic circulation—where the arterial pressure is approximately six times higher than that of the pulmonary system—any vascular rupture involving these vessels or non-bronchial systemic arteries (NBSAs) is uniquely prone to inducing massive hemoptysis, accounting for nearly 90% of clinical cases (12). For this reason, endovascular interventional therapy for hemoptysis centers predominantly on the selective embolization of these culprit bronchial arteries or NBSAs.

### 4.2 Application anatomy of interventional embolization therapy and selection of embolic materials

The bronchial arteries frequently share a common trunk with the ipsilateral intercostal arteries (intercostobronchial trunks), which may give rise to critical radiculomedullary or spinal branches. Inadvertent nontarget embolization of these collateral vessels can precipitate catastrophic neurological complications, including spinal cord infarction or permanent paraplegia. Consequently, meticulous pre-embolization angiography to precisely delineate the vascular anatomy and identify the hairpin course of the anterior spinal artery is paramount (13). This anatomical hazard accounts for the technical failure encountered during the second intervention for Patient 14, where a spinal branch originated from the culprit intercostobronchial trunk, and the microcatheter could not safely bypass the orifice of the spinal artery to reach the target bronchial branches. To mitigate the imminent risk of spinal cord ischemia from embolic reflux, the procedure was prudently aborted, and the patient was successfully managed with surgical resection (Figure 1).

**Figure 1:**
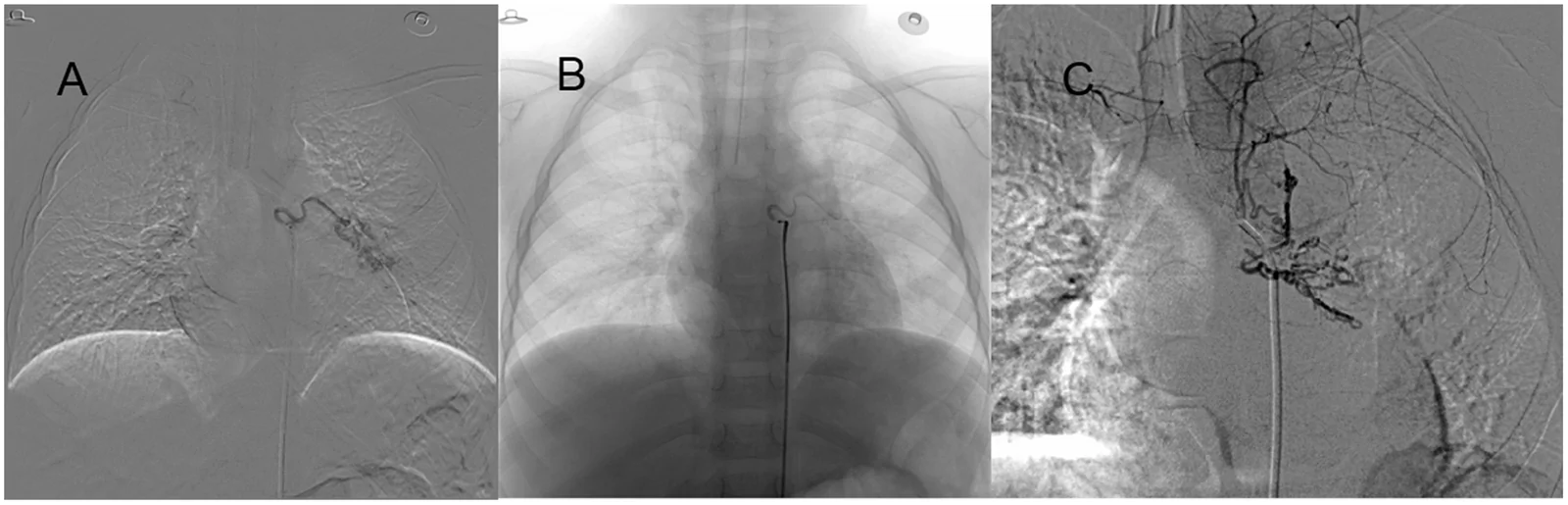
Angiographic findings and procedural challenges in patient 14. **(A)** Digital subtraction angiography (DSA) during the initial intervention showing the left bronchial artery before embolization. **(B)** Position of the microcatheter at the proximal/initial segment of the left bronchial artery during the first embolization procedure. **(C)** Repeat angiography performed 2 months later due to recurrent hemoptysis, revealing that the culprit bronchial artery originated directly from the ipsilateral spinal artery; the severe vascular tortuosity and slender caliber of the vessel precluded super-selective catheterization with the microcatheter.

Ideally, the catheter should bypass redundant side branches and be super-selectively advanced into the distal segment of the target vessel for embolization, thereby preventing inadvertent nontarget occlusion of other major arterial structures. Currently, particulate or microspherical embolic agents represent the mainstay of frontline clinical practice, whereas metallic coils are generally not utilized as standalone embolic materials (14). The selection of specific embolic particle sizes depends heavily on the morphologic variability of the target vessel and the internal diameter of the catheter deployed (15). In the current study, intraoperative angiography invariably demonstrated early contrast opacification of the pulmonary arteries in all 17 cases, confirming the presence of BPAFs. In the presence of such shunts, deployment of excessively small embolic particles poses an imminent risk of migration through the abnormal bronchopulmonary anastomoses, potentially culminating in non-target pulmonary or systemic arterial embolism. Literature indicates that the internal diameter of these bronchopulmonary shunts is generally less than or equal to 325*μ*m (16), and no statistically significant difference in clinical efficacy has been established between 300 and 500 μm and 500-700 μm particle sizes (17). Therefore, 300-500 μm PVA particles were chosen as the primary embolic medium in this study. Notably, for Patients 11 and 13, who presented with markedly dilated, large-caliber bronchial arteries, gelatin sponge particles were adjunctively deployed to consolidate the embolization effect and achieve secure vascular occlusion.

### 4.3 Efficacy and complications of interventional embolization therapy

The reported success rate of transcatheter embolization for hemoptysis generally ranges from 73% to 99% (18). Clinical recurrence is typically attributed to the recanalization of previously embolized target vessels or the progression of primary diseases inciting neo-collateralization. In our cohort, acute hemoptysis was successfully controlled within 5 days post-procedure in 16 patients. However, three patients experienced recurrent bleeding during the long-term follow-up. This recurrence was likely associated with the unique hemodynamic and anatomical characteristics of their BPAFs, which featured high-flow shunts, multi-focal feeder origins, and marked vascular tortuosity.

Patient 17 presented with severe pulmonary venous congestion secondary to congenital pulmonary hypoplasia, the definitive cause of his hemoptysis. Targeted bronchial artery embolization alone was pathophysiologically inadequate to control bleeding, and curative resection was required thereafter. All participants received postoperative follow-up for at least one year as scheduled. The recurrence in Patient 17 emerged later than expected, representing a well-recognized limitation of this longitudinal observational study.

Common complications associated with transcatheter embolization for hemoptysis include transient dysphagia and chest pain, with reported incidence rates of 1% to 18% and 24% to 91%, respectively (19). These adverse events are typically secondary to the non-target embolization of the esophageal and intercostal arteries. More catastrophically, permanent paraplegia and spinal cord ischemia represent severe complications linked to the inadvertent occlusion of spinal feeding branches (20). Literature indicates that embolic microspheres or particles with a diameter ≥100μm are size-excluded from entering these critical spinal arterial branches (21). In the current study, because the microcatheter was meticulously navigated to bypass redundant collateral branches and the embolic size was strictly restricted to 300-500μm, no intraoperative or postoperative ischemic neurological complications or systemic adverse events were observed. Coils were reserved for specific anatomical situations such as a dilated main trunk, high-flow systemic-to-pulmonary shunting, or pseudoaneurysm. However, the bronchial arteries in pediatric patients are generally small in caliber. Coils alone can only address the main bronchial artery trunk and are insufficient for managing bronchial artery-to-pulmonary artery fistulas, yielding suboptimal hemostatic efficacy. Moreover, in the event of recurrent hemoptysis, retained coils may compromise subsequent interventional procedures.

## 5 Limitation

Nevertheless, several limitations of this study must be acknowledged. First, this was a single-center retrospective investigation involving a small cohort of 17 patients, which restricts the external validity and generalizability of our findings. Second, from a safety perspective, although no procedural complications occurred in the present cohort, this zero-incidence observation is insufficient to definitively guarantee absolute safety; rare but catastrophic adverse events, such as spinal cord ischemia, may simply evade detection within such a small sample size. Lastly, the standardized post-procedure follow-up was restricted to 12 months, precluding an assessment of the long-term risk of hemoptysis recurrence. Consequently, large-scale, multi-center prospective randomized trials with extended follow-up periods and systematic safety evaluations remain imperative to further substantiate the long-term safety, durability, and efficacy of interventional management for pediatric BPAF-related hemoptysis.

## 6 Conclusion

Transcatheter interventional embolization is a safe and effective therapeutic modality for managing hemoptysis secondary to BPAFs in pediatric patients, demonstrating high clinical efficacy and a low complication rate. For complex BPAF cases characterized by high-volume shunts, multi-stage or recurrent interventional procedures may be required, necessitating the meticulous selection of embolic particle diameters. Furthermore, in cases involving highly tortuous bronchial arteries, super-selective advancement of the microcatheter as close to the fistula orifice as technically feasible is paramount to maximize secure vascular occlusion and optimize therapeutic outcomes. Considering the relatively small sample size, the present results should be interpreted with caution and verified by larger follow-up studies.

## Data Availability

The original contributions presented in the study are included in the article/Supplementary Material, further inquiries can be directed to the corresponding author.
